# Nephroprotective Effect of Mesenchymal Stem Cell-Based Therapy of Kidney Disease Induced by Toxicants

**DOI:** 10.1155/2020/8819757

**Published:** 2020-12-21

**Authors:** Shujun Lin, Wenshan Lin, Chunling Liao, Tianbiao Zhou

**Affiliations:** Department of Nephrology, The Second Affiliated Hospital, Shantou University Medical College, 515041 Shantou, China

## Abstract

**Background:**

Renal damage caused by drug toxicity is becoming increasingly common in the clinic. Preventing and treating kidney damage caused by drug toxicity are essential to maintain patient health and reduce the social and economic burden. In this study, we performed a meta-analysis to assess the nephroprotective effect of mesenchymal stem cells (MSCs) in the treatment of kidney disease induced by toxicants.

**Methods:**

The Cochrane Library, Embase, ISI Web of Science, and PubMed databases were searched up to December 31, 2019, to identify studies and extract data to assess the efficacy of MSCs treatment of kidney disease induced by toxicants using Cochrane Review Manager Version 5.3. A total of 27 studies were eligible and selected for this meta-analysis.

**Results:**

The results showed that a difference in serum creatinine levels between the MSC treatment group and control group was observed for 2, 4, 5, 6-8, 10-15, 28-30, and ≥42 days (2 days: WMD = −0.88, 95% CI: -1.34, -0.42, *P* = 0.0002; 4 days: WMD = −0.74, 95% CI: -0.95, -0.54, *P* < 0.00001; 5 days: WMD = −0.46, 95% CI: -0.67, -0.25, *P* < 0.0001; 6-8 days: WMD = −0.55, 95% CI: -0.84, -0.26, *P* = 0.0002; 10-15 days: WMD = −0.37, 95% CI: -0.53, -0.20, *P* < 0.0001; 28-30 days: WMD = −0.53, 95% CI: -1.04, -0.02, *P* = 0.04; ≥42 days: WMD = −0.22, 95% CI: -0.39, -0.06, *P* = 0.007). Furthermore, a difference in blood urea nitrogen levels between the MSC treatment group and control group was observed for 2-3, 4-5, 6-8, and ≥28 days. The results also indicate that MSC treatment alleviated inflammatory cells, necrotic tubules, regenerative tubules, and renal interstitial fibrosis in kidney disease induced by toxicants.

**Conclusion:**

MSCs may be a promising therapeutic agent for kidney disease induced by toxicants.

## 1. Introduction

Kidney injury occurs during acute kidney injury (AKI) and chronic kidney disease (CKD), and it is a common condition associated with the morbidity and mortality of patients. A total of 80% of patients who survive an AKI episode completely recover kidney function, and recovered AKI patients present an almost 9-fold increase in risk for CKD development [1]. Toxicant-induced kidney injury is one of the most common causes of kidney disease, causing substantial morbidity and hampering drug development [2]. At present, renal damage caused by drug toxicity is becoming increasingly common in the clinic. Preventing and treating kidney damage caused by drug toxicity is essential to maintain patient health and reduce the social and economic burden.

Mesenchymal stem cells (MSCs), which are multipotent mesenchymal cells present in various tissues, have multilineage differentiation ability under appropriate conditions and are easy to obtain. They are a promising therapeutic option for some diseases because of their unique property of releasing some important bioactive factors [3–5]. Drug toxicity can induce renal tubular epithelial cell damage or death and can lead to renal interstitial inflammation, which eventually develops into renal interstitial fibrosis and renal loss. Previous studies have shown that MSCs can play a protective role against injury of renal tubular epithelial cells and prevent renal interstitial fibrosis [6–10]. Before clinical application, animal experiments in vivo are generally required to confirm the effectiveness of MSCs. Furthermore, there are few clinical trials of MSCs on kidney disease induced by toxicants. Therefore, in this study, we performed a meta-analysis to assess the nephroprotective effect of MSCs in the treatment of kidney disease induced by toxicants in animals.

## 2. Materials and Methods

### 2.1. Search Strategy

We searched databases (Cochrane Library, Embase, ISI Web of Science, and PubMed) up to Dec 31, 2019, using the following search terms: (mesenchymal stem cells OR MSC OR MSCs OR multipotent stromal cells OR mesenchymal stromal cells OR mesenchymal progenitor cells OR stem cells) AND (gentamicin OR aristolochic acid OR cisplatin OR adriamycin OR cadmium chloride OR methotrexate OR rifampicin OR glycerol OR streptozocin) AND (kidney injury OR renal failure OR kidney disease). The search was confined to English-language literature. An additional search was conducted among the eligible manual references of the cited articles.

### 2.2. Inclusion and Exclusion Criteria

Our meta-analysis included studies analyzing the efficacy of MSC treatment in mice or rats with kidney disease. The following studies were excluded from the analysis: (1) letters, case reports, reviews, clinical studies, editorials, meta-analysis, and systematic reviews; (2) studies lacking the targeted indicators or number of case or control groups and were conducted in humans; (3) studies of kidney disease that was not induced by toxicants; and (4) studies with therapeutic regimen for kidney disease that included other agents with undefined effects.

### 2.3. Outcome Measures

We filtered the following outcomes associated with the efficacy of MSC treatment from the recruited studies: serum creatinine (Scr), blood urea nitrogen (BUN), urinary albumin excretion (UAE), malondialdehyde (MDA), L-glutathione (GSH), superoxide dismutase (SOD), and renal pathology. In addition, we conducted a mutual consensus when met with disagreements.

### 2.4. Quality Assessment

Two investigators independently evaluated the methodological quality using the Cochrane Handbook for Interventions. We assessed the following sections of every investigation: selection bias, attrition bias, performance bias, detection bias, reporting bias, and other bias. Each item was classified as unclear, high risk, or low risk.

### 2.5. Statistical Analysis

Review Manager Version 5.3 was applied to explore whether MSC treatment achieved a good efficacy in kidney disease induced by toxicants, and STATA 12.0 was used to test the publication bias. Heterogeneity of variation among individual studies was quantified and described using *I*^2^. The fixed effects model was used if the *P* value of the heterogeneity test was ≥ 0.1. Otherwise, the random effects model was applied to pool the outcomes. In addition, to compute continuous variables, we analyzed weighted mean differences (WMDs) for the mean values. We also calculated 95% confidence intervals (95% CI) for the included studies using the Mantel-Haenszel (M-H) method. Additionally, we evaluated the publication bias using Begg's rank correlation test as well as Egger's linear regression method among the studies. A *P* value < 0.05 was considered of statistical significance.

## 3. Results

### 3.1. Search Results

**Figure 1:**
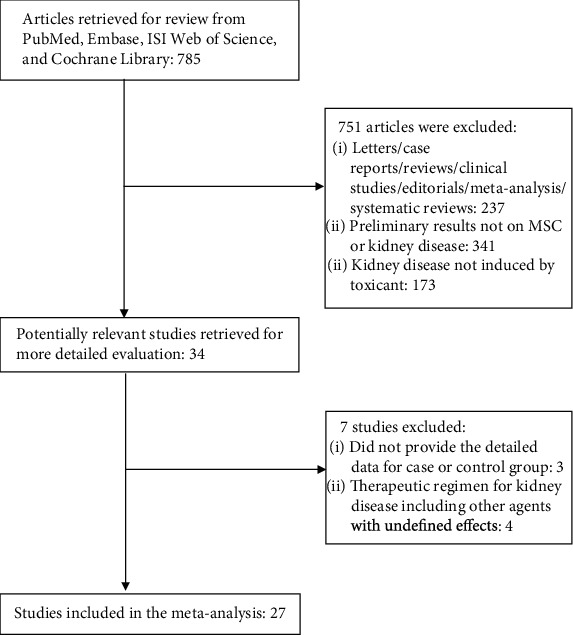
Flow diagram of the selection process.

The databases mentioned above were searched, and only studies in mice or rats that evaluated the therapeutic efficacy of MSC treatment on kidney disease induced by toxicants were selected. Twenty-seven studies [11–37] were eligible and selected for this meta-analysis, and a flowchart of inclusion of studies is presented in Figure 1. Study characteristics are shown in Table 1.

### 3.2. Quality Assessment of Included Studies

**Figure 2:**
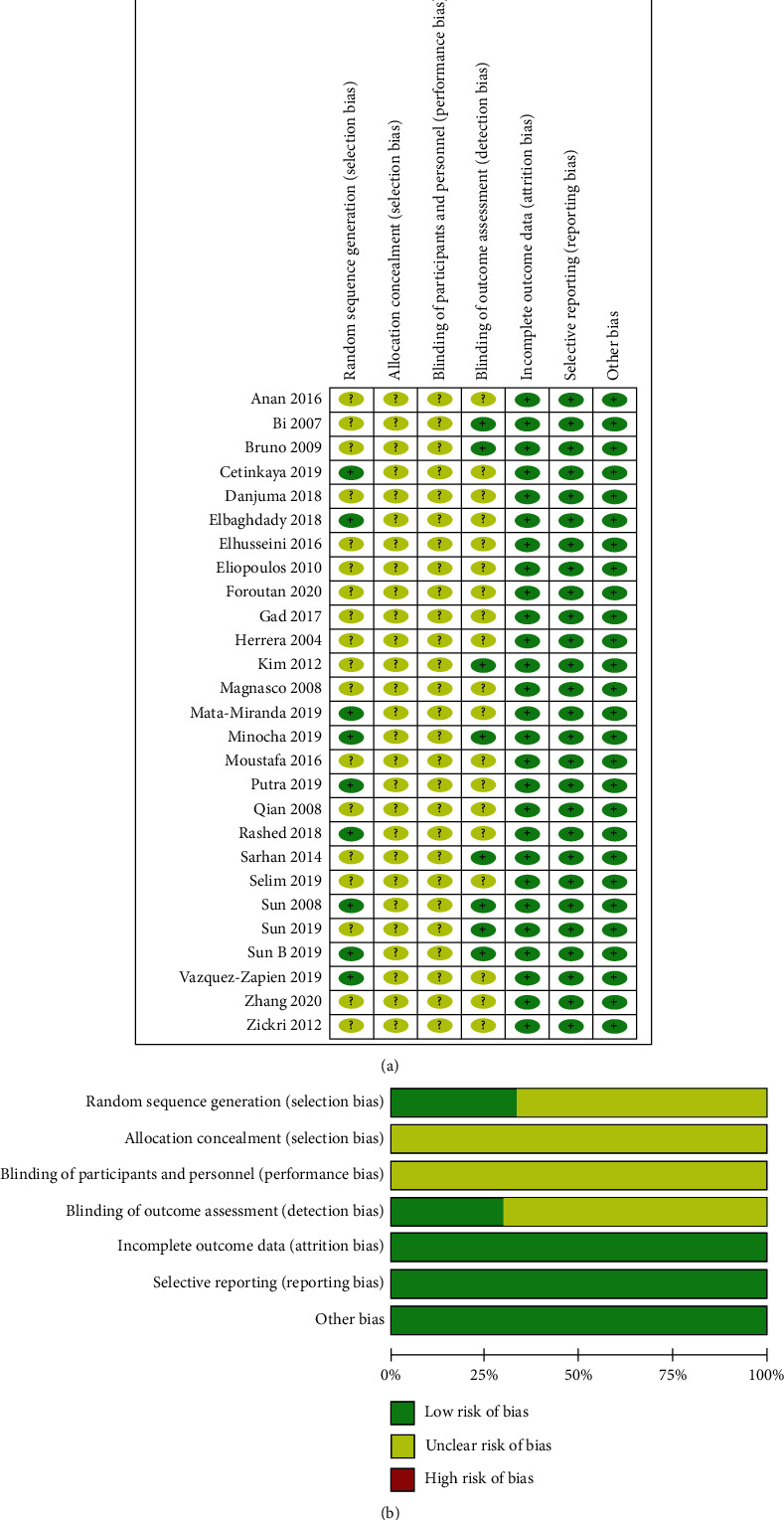
(a) Aggregate Risk of bias graph for each experimental animal studies; “?”: Unclear risk; “+”: Low risk. (b) Risk of bias summary.

The methodological quality of the selected studies was considered acceptable because most study domains were ranked as unclear risk or low risk of bias. Unclear risk of bias was mostly detected in performance and selection bias. Low risk of bias mostly occurred in detection, reporting, and attrition bias.  Figure 2 shows a summary of the risk of biases of the selected studies.

### 3.3. Scr

**Figure 3:**
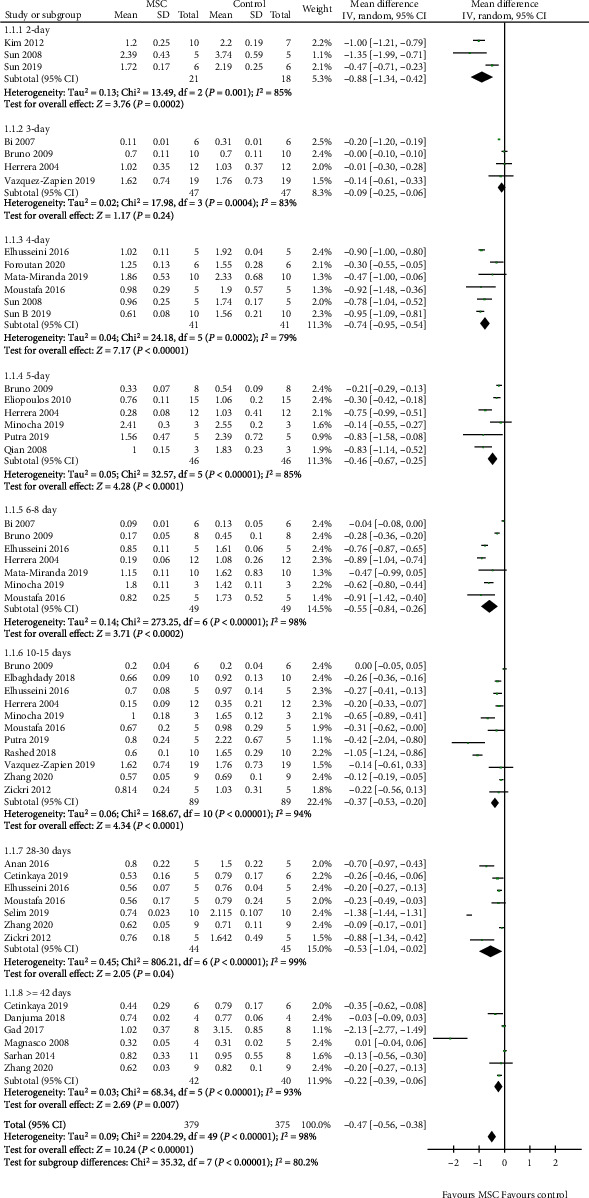
Effect of MSC on Scr.

A total of 27 studies [11–37] were selected to assess the effect of MSCs on Scr, and the results show that a difference between the MSC treatment and control groups was observed for 2, 4, 5, 6-8, 10-15, 28-30 days, and ≥42 days (2 days: WMD = −0.88, 95% CI: -1.34, -0.42, *P* = 0.0002; 4 days: WMD = −0.74, 95% CI: -0.95, -0.54, *P* < 0.00001; 5 days: WMD = −0.46, 95% CI: -0.67, -0.25, *P* < 0.0001; 6-8 days: WMD = −0.55, 95% CI: -0.84, -0.26, *P* = 0.0002; 10-15 days: WMD = −0.37, 95% CI: -0.53, -0.20, *P* < 0.0001; 28-30 days: WMD = −0.53, 95% CI: -1.04, -0.02, *P* = 0.04; ≥42 days: WMD = −0.22, 95% CI: -0.39, -0.06, *P* = 0.007; Figure 3 and Table 2). However, no difference was observed between the MSC treatment and control groups for 3 days (3 days: WMD = −0.09, 95% CI: -0.25, -0.06, *P* = 0.24; Figure 3 and Table 2).

### 3.4. BUN

**Figure 4:**
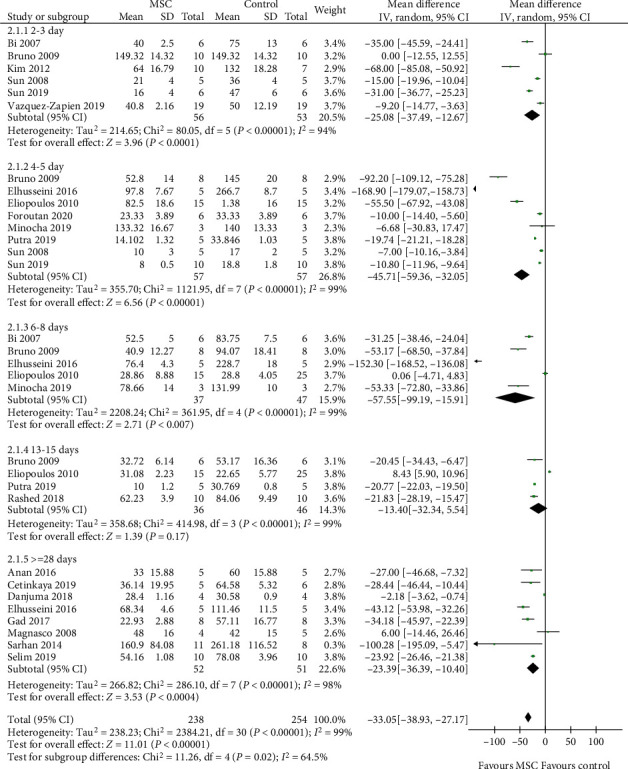
Effect of MSC on BUN.

A total of 18 studies [11–15, 17–19, 21, 22, 24, 26–29, 32–34, 36, 37] were selected to assess the effect of MSCs on BUN, and the results indicate that the difference between the MSC treatment and control groups was observed for 2-3, 4-5, 6-8, and ≥28 days (2-3 days: WMD = −25.08, 95% CI: -37.49, -12.67, *P* < 0.0001; 4-5 days: WMD = −45.71, 95% CI: -59.36, -32.05, *P* < 0.00001; 6-8 days: WMD = −57.55, 95% CI: -99.19, -15.91, *P* = 0.007; ≥28 days: WMD = −23.39, 95% CI: -36.39, -10.40, *P* = 0.0004; Figure 4 and Table 2). However, no difference was observed between the MSC treatment and control groups for 13-15 days (WMD = −13.40, 95% CI: -32.34, 5.54, *P* = 0.17; Figure 4 and Table 2).

### 3.5. Urinary Albumin Excretion

Three studies [22, 26, 27] were selected in the meta-analysis for the assessment of MSCs on UAE. The results show that the MSC group had a lower UAE than the control group (WMD = −22.66, 95% CI: -26.41, -18.90, *P* < 0.00001; Table 2).

### 3.6. Oxidative Stress

Four studies [17, 19, 23, 27] were selected for the assessment of MDA, four [17, 19, 23, 27] for GSH, and three [11, 17, 23] for SOD. The results indicate that a difference between the MSC treatment and control groups was observed for MDA, GSH, and SOD (MDA: WMD = −17.21, 95% CI: -20.38, -14.04, *P* < 0.00001; GSH: WMD = 4.62, 95% CI: 2.74, 6.50, *P* < 0.00001; SOD: WMD = 5.42, 95% CI: 2.92, 7.93, *P* < 0.0001; Table 2).

### 3.7. Assessment of Renal Pathology

Four studies [17, 24, 27, 35] for inflammatory cells, two studies [17, 27] for necrotic tubules, two studies [17, 27] for regenerative tubules, and three studies [17, 27, 35] for renal interstitial fibrosis were included in this meta-analysis. The results indicate that the difference in inflammatory cells, necrotic tubules, regenerative tubules, and renal interstitial fibrosis between the MSC treatment and control groups was significant (inflammatory cells: WMD = −2.66, 95% CI: -3.83, -1.49, *P* < 0.00001; necrotic tubules: WMD = −2.58, 95% CI: -4.75, -0.40, *P* = 0.02; regenerative tubules: WMD = 6.00, 95% CI: 3.45, 8.55, *P* < 0.00001; renal interstitial fibrosis: WMD = −5.82, 95% CI: -7.41, -4.23, *P* < 0.00001; Table 2).

### 3.8. Publication Bias

**Figure 5:**
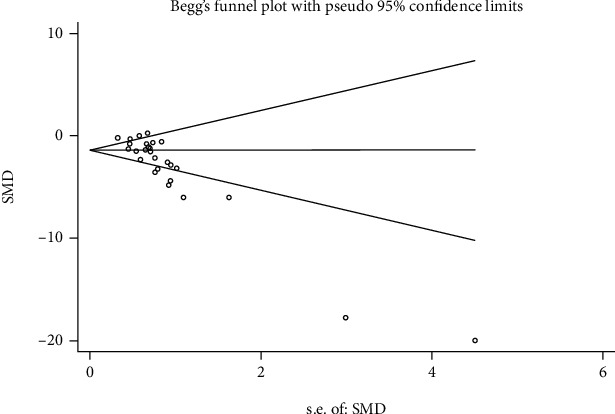
Publication bias.

Publication bias was tested in this meta-analysis, and a funnel plot generated using STATA 12.0 for the primary outcome. Begg's test and Egger's test results suggest that publication bias was present (*P* ≤ 0.01 and *P* ≤ 0.01, respectively; Figure 5).

## 4. Discussion

We reviewed all the selected studies and evaluated the Scr, BUN, UAE, oxidative stress, and renal pathology results to assess the nephroprotective effect of MSCs in the treatment of kidney disease induced by toxicants. We found that MSC treatment reduced Scr levels at 2, 4, 5, 6-8, 10-15, 28-30, and ≥42 days and reduced BUN levels at 2-3, 4-5, 6-8, and ≥28 days. We also found that the MSC group had a lower UAE than the control group. It has been previously shown that MSC treatment reduces the levels of Scr, BUN, and proteinuria in lupus nephritis in mice [38]. Chen et al. [39] found that MSC ameliorates ischemia/reperfusion injury-induced acute kidney injury in rats and reduces Scr levels. Xiu et al. [40] found that MSC transplantation significantly reduces the concentration of BUN and Scr, prevents tissue injury, and reduces mortality after lipopolysaccharide-induced acute kidney injury. Clinical trials also supported that MSC injection decreases rejection after transplantation. Tan et al. [41] found that the therapy with MSCs achieve better renal function and lower incidence of acute rejection at 1 year compared with the anti-IL-2 receptor antibody induction. Vanikar et al. [42] demonstrated that infusion of MSCs as well as hematopoietic stem cells eases immunosuppression in living donor renal transplantation. Our previous meta-analysis also found that MSCs reduce Scr levels, BUN levels, and proteinuria, as well as alleviate renal damage in animal models of AKI [43]. Lower proteinuria was also found in patients with SLE after MSC therapy [44].

The MSC treatment group had a higher level of GSH, SOD, and a lower level of MDA when compared with the control group. El-Metwaly et al. [45] found that MSCs increase GSH levels and reduce MDA levels in lung tissue of rats subjected to acute lung injury. Li et al. [46] reported that MSCs can restore the levels of GSH and MDA in rats with chronic alcoholism, and its effects on repairing sciatic nerve were obvious. Liu et al. [47] reported that MSCs significantly increase the activity of glutathione (GSH) and reduce the levels of MDA in rats induced by unilateral ureteral obstruction.

The mechanism by which MSCs repair injured kidneys may be complex. After kidney injury, VCAM-1, GFP, SDF -1/CXCR4, and CD44 are upregulated in the injured tissue, which may play important roles in the migration of MSCs to the damaged area. These substances may be partly secreted by the MSCs themselves [20, 48, 49]. The presence of MSCs may limit the injury and repair the ischemic tubular damage to maintain the glomerular filtration rate and downregulate BUN [50]. In addition, MSCs lower the expression of several proinflammatory cytokines such as TNF-*α*, IL-1*β*, and IFN-*γ* as well as increase anti-inflammatory cytokines such as IL-1, IL-10, Bcl-2, TNF-*α*, bFGF, and prostaglandin E2 [49, 51]. Another possibility is that MSCs may restore damaged cells and prevent apoptosis by secreting microvesicles, which contain microRNAs, mRNAs, or proteins [49]. To conclude, MSCs can migrate to the damaged tissue, promote the recovery of renal function, enhance proliferation, and reduce fibrosis and inflammation.

Furthermore, our study indicates that MSC treatment can alleviate inflammatory cells, necrotic tubules, regenerative tubules, and renal interstitial fibrosis in kidney disease induced by toxicants. Some previous studies indicated that MSC treatment can alleviate renal pathological changes in unilateral ureteral obstruction rat or mice [9, 10, 52].

However, this meta-analysis also has some limitations. First, a small sample size was found for the recruited studies. The administered dose and the type of MSCs were not exactly the same. Publication bias was found in this meta-analysis, and the results should be reassessed in the future. Furthermore, the studies frequently had different animal models (mouse or rat), toxin doses, and administration routes for renal injury. These limitations may affect the robustness of our results.

## 5. Conclusions

The MSC treatment reduced Scr levels after 2, 4, 5, 6-8, 10-15, 28-30, and ≥42 days and reduced BUN levels after 2-3, 4-5, 6-8, and ≥28 days. The results also indicate that MSC treatment alleviated the inflammatory cells, necrotic tubules, regenerative tubules, and renal interstitial fibrosis in kidney disease induced by toxicants.

## Figures and Tables

**Table 1:** Characteristics of the studies included in this meta-analysis.

Author, year	*n*	Type of animal	Type of injury	MSC type	Number of MSC	Route of delivery	Endpoints for this meta-analysis
Herrera 2004	24	Mice	Glycerol-induced	BM-MSCs	1 × 10^6^	Intravenous injection	Scr
Bi 2007	12	Mice	Cisplatin-induced	BM-MSCs	2 × 10^5^	Intravenous injection or intraperitoneal injection	Scr, BUN
Sun 2008	40	Rat	Glycerol-induced	BM-MSCs	2 × 10^6^	Abdominal aorta injection	Scr, BUN
Qian 2008	6	Rat	Glycerol-induced	BM-MSCs	1 × 10^4^	Intravenous injection	Scr
Magnasco 2008	22	Rat	Adriamycin-induced	BM-MSCs	3 × 10^6^	Intravenous injection	Scr, BUN, UAE, renal damage score
Bruno 2009	16	Mice	Glycerol-induced	BM-MSCs	—	Intravenous injection	Scr, BUN, MDA, GSH, SOD, renal damage score
Eliopoulos 2010	10	Mice	Cisplatin-induced	BM-MSCs	5 × 10^6^	Intraperitoneal injection	Scr, BUN
Kim 2012	17	Rat	Cisplatin-induced	AD-MSCs	5 × 10^5^	Intravenous injection	Scr, BUN
Zickri 2012	30	Rat	Adriamycin-induced	hUC-MSCs	5 × 10^5^	Intravenous injection	Scr
Sarhan 2014	19	Rat	Adriamycin-induced	BM-MSCs	4 × 10^6^	Intravenous injection	Scr, BUN, UAE, renal pathology, MDA, GSH
Moustafa 2016	80	Rat	Cisplatin-induced	BM-MSCs	5 × 10^6^	Intravenous, intraarterial or kidney subcapsular injection	Scr, MDA, GSH, SOD
Elhusseini 2016	40	Rat	Cisplatin-induced	AD-MSCs	5 × 10^6^	Intravenous injection	Scr, BUN, Ccr, renal pathology, MDA, GSH, SOD
Anan 2016	13	Rat	Adriamycin-induced	BM-MSCs	1 × 10^6^	Intravenous injection	Scr, BUN, SOD
Gad 2017	24	Rat	Methotrexate-induced	BM-MSCs	2 × 10^6^	Intraperitoneal injection	Scr, BUN, MDA, GSH
Rashed 2018	20	Rat	Streptozotocin -induced	BM-MSCs	1 × 10^6^	Intravenous injection	Scr, BUN, UAE, Ccr
Elbaghdady 2018	20	Rat	Cadmium chloride-induced	BM-MSCs	2 × 10^6^	Intravenous injection	Scr
Danjuma 2018	16	Rat	Rifampicin-induced	BM-MSCs	2.5 × 10^5^	Intravenous injection	Scr, BUN
Putra 2019	10	Rat	Gentamicin induced	hUC-MSCs	1 × 10^6^	Intraperitoneal injection	Scr, BUN, renal pathology
Cetinkaya 2019	17	Rat	Aristolochic acid induced	hAMSC	6 × 10^5^	Intravenous injection	Scr, BUN
Selim 2019	70	Rat	Cisplatin-induced	AD-MSCs; BM-MSCs	4 × 10^6^	Intravenous injection	Scr, BUN
Mata-Miranda 2019	10	Mice	Cisplatin-induced	mESCs	1 × 10^6^	Intraperitoneal injection	Scr
Vazquez-Zapien 2019	19	Mice	Cisplatin-induced	mESCs	1 × 10^6^	Intraperitoneal injection	Scr
Minocha 2019	3	Rat	Cisplatin-induced	AFSC	2 × 10^6^	Intravenous injection	Scr, BUN
Sun B 2019	10	Rat	Cisplatin-induced	USCs	2 × 10^6^	Intravenous injection	Scr
Sun 2019	6	Rat	Cisplatin-induced	BM-MSCs	—	Renal parenchyma injection	Scr, BUN
Zhang 2020	9	Rat	Cisplatin-induced	USCs	5 × 10^6^	Subcutaneous injection	Scr, Ccr, renal pathology
Foroutan 2020	6	Rat	Cisplatin-induced	BM-MSCs	—	Intraperitoneal injection	Scr, BUN

Note: BM-MSCs: bone marrow mesenchymal stem cells; hAMSCs: human amnion-derived mesenchymal stem cells; hUC-MSCs: human umbilical cord-mesenchymal stem cells; AD-MSCs: adipose tissue-derived mesenchymal stem cells; mESCs: mouse embryonic stem cells; AFSCs: amniotic fluid stem cells; USCs: urine-derived stem cells; Scr: serum creatinine; BUN: blood urea nitrogen; UAE: urinary albumin excretion; Ccr: creatinine clearance rate; MDA: malondialdehyde; GSH: L-glutathione; SOD: superoxide dismutase.

**Table 2:** Meta-analysis of the efficacy of MSC in therapy of renal injury induced by toxicant.

Indicators	Time point	Studies	*Q* test	Model	OR/WMD	*P*
		Number	*P* value	selected	(95% CI)	
Scr	2 days	3	0.001	Random	-0.88 (-1.34, -0.42)	0.0002
	3 days	4	0.0004	Random	-0.09 (-0.25, 0.06)	0.24
	4 days	6	0.0002	Random	-0.74 (-0.95, -0.54)	<0.00001
	5 days	6	<0.00001	Random	-0.46 (-0.67, -0.25)	<0.0001
	6-8 days	7	<0.00001	Random	-0.55 (-0.84, -0.26)	0.0002
	10-15 days	11	<0.00001	Random	-0.37 (-0.53, -0.20)	<0.0001
	28-30 days	7	<0.00001	Random	-0.53 (-1.04, -0.02)	0.04
	≥42 days	6	<0.00001	Random	-0.22 (-0.39, -0.06)	0.007
BUN	2-3 days	6	<0.00001	Random	-25.08 (-37.49, -12.67)	<0.0001
	4-5 days	8	<0.00001	Random	-45.71 (-59.36, -32.05)	<0.00001
	6-8 days	5	<0.00001	Random	-57.55 (-99.19, -15.91)	0.007
	13-15 days	4	<0.00001	Random	-13.40 (-32.34, 5.54)	0.17
	≥28 days	8	<0.00001	Random	-23.39 (-36.39, -10.40)	0.0004
UAE	—	3	0.72	Fixed	-22.66 (-26.41, -18.90)	<0.00001
MDA	—	4	0.41	Fixed	-17.21 (-20.38, -14.04)	<0.00001
GSH	—	4	<0.00001	Random	4.62 (2.74, 6.50)	<0.00001
SOD	—	3	<0.00001	Random	5.42 (2.92, 7.93)	<0.0001
Renal pathology						
Inflammatory cells	—	4	<0.00001	Random	-2.66 (-3.83, -1.49)	<0.00001
Necrotic tubule	—	2	<0.00001	Random	-2.58 (-4.75, -0.40)	0.02
Regenerative tubules	—	2	—	Fixed	6.00 (3.45, 8.55)	<0.00001
Renal interstitial fibrosis	—	3	<0.00001	Random	-5.82 (-7.41, -4.23)	<0.00001

Note: Scr: serum creatinine; BUN: blood urea nitrogen; UAE: urinary albumin excretion; Ccr: creatinine clearance rate; MDA: malondialdehyde; GSH: L-glutathione; SOD: superoxide dismutase.

## Data Availability

The data supporting this meta-analysis are from previously reported studies and datasets, which have been cited. The processed data are available from the corresponding author upon request.

## Abbreviations

MSCs: Mesenchymal stem cells
Scr: Serum creatinine
BUN: Blood urea nitrogen
UAE: Urinary albumin excretion
MDA: Malondialdehyde
GSH: L-glutathione
SOD: Superoxide dismutase
WMDs: Weighted mean differences
CI: Confidence intervals
M-H: Mantel-Haenszel.

## References

[1] Basile D. P., Bonventre J. V., Mehta R. (2016). Progression after AKI: understanding maladaptive repair processes to predict and identify therapeutic treatments. *Journal of the American Society of Nephrology*.

[2] Pianta T. J., Buckley N. A., Peake P. W., Endre Z. H. (2013). Clinical use of biomarkers for toxicant-induced acute kidney injury. *Biomarkers in Medicine*.

[3] Liang Y., Zhang D., Li L. (2020). Exosomal microRNA-144 from bone marrow-derived mesenchymal stem cells inhibits the progression of non-small cell lung cancer by targeting CCNE1 and CCNE2. *Stem Cell Research & Therapy*.

[4] Ferrari E., Soloviev M., Jasmin (2020). In vitro labeling mesenchymal stem cells with superparamagnetic iron oxide nanoparticles: efficacy and cytotoxicity. *Nanoparticles in Biology and Medicine*.

[5] Zhu Y., Zhang X., Gu R. (2020). LAMA2 regulates the fate commitment of mesenchymal stem cells via hedgehog signaling. *Stem Cell Research & Therapy*.

[6] He J., Jiang Y. L., Wang Y., Tian X. J., Sun S. R. (2020). Micro-vesicles from mesenchymal stem cells over-expressing miR-34a inhibit transforming growth factor-*β*1-induced epithelial-mesenchymal transition in renal tubular epithelial cells in vitro. *Chinese Medical Journal*.

[7] Li D., Zhang D., Tang B. (2019). Exosomes from human umbilical cord mesenchymal stem cells reduce damage from oxidative stress and the epithelial-mesenchymal transition in renal epithelial cells exposed to oxalate and calcium oxalate monohydrate. *Stem Cells International*.

[8] Liu B., Ding F., Hu D. (2018). Human umbilical cord mesenchymal stem cell conditioned medium attenuates renal fibrosis by reducing inflammation and epithelial-to-mesenchymal transition via the TLR4/NF-*κ*B signaling pathway in vivo and in vitro. *Stem Cell Research & Therapy*.

[9] Xing L., Song E., Yu C. Y. (2018). Bone marrow-derived mesenchymal stem cells attenuate tubulointerstitial injury through multiple mechanisms in UUO model. *Journal of Cellular Biochemistry*.

[10] Zheng J., Wang Q., Leng W., Sun X., Peng J. (2018). Bone marrow‑derived mesenchymal stem cell‑conditioned medium attenuates tubulointerstitial fibrosis by inhibiting monocyte mobilization in an irreversible model of unilateral ureteral obstruction. *Molecular Medicine Reports*.

[11] Anan H. H., Zidan R. A., Shaheen M. A., Abd- el Fattah E. A. (2016). Therapeutic efficacy of bone marrow derived mesenchymal stromal cells versus losartan on adriamycin-induced renal cortical injury in adult albino rats. *Cytotherapy*.

[12] Bi B., Schmitt R., Israilova M., Nishio H., Cantley L. G. (2007). Stromal cells protect against acute tubular Injuryviaan endocrine effect. *Journal of the American Society of Nephrology*.

[13] Bruno S., Grange C., Deregibus M. C. (2009). Mesenchymal stem cell-derived microvesicles protect against acute tubular injury. *Journal of the American Society of Nephrology*.

[14] Cetinkaya B., Unek G., Kipmen-Korgun D., Koksoy S., Korgun E. T. (2019). Effects of human placental amnion derived mesenchymal stem cells on proliferation and apoptosis mechanisms in chronic kidney disease in the rat. *International Journal of Stem Cells*.

[15] Danjuma L., Mok P. L., Higuchi A. (2018). Modulatory and regenerative potential of transplanted bone marrow-derived mesenchymal stem cells on rifampicin-induced kidney toxicity. *Regenerative Therapy*.

[16] Elbaghdady H. A. M., Alwaili M. A., El-Demerdash R. S. (2018). Regenerative potential of bone marrow mesenchymal stem cells on cadmium chloride-induced hepato-renal injury and testicular dysfunction in sprague dawley rats. *Ecotoxicology and Environmental Safety*.

[17] Elhusseini F. M., Saad M. A., Anber N. (2016). Long term study of protective mechanisms of human adipose derived mesenchymal stem cells on cisplatin induced kidney injury in Sprague-Daweley rats. *Journal of Stem Cells and Regenerative Medicine*.

[18] Eliopoulos N., Zhao J., Bouchentouf M. (2010). Human marrow-derived mesenchymal stromal cells decrease cisplatin renotoxicity in vitro and in vivo and enhance survival of mice post-intraperitoneal injection. *American Journal of Physiology-Renal Physiology*.

[19] Gad A. M., Hassan W. A., Fikry E. M. (2017). Significant curative functions of the mesenchymal stem cells on methotrexate-induced kidney and liver injuries in rats. *Journal of Biochemical and Molecular Toxicology*.

[20] Herrera M. B., Bussolati B., Bruno S., Fonsato V., Romanazzi G. M., Camussi G. (2004). Mesenchymal stem cells contribute to the renal repair of acute tubular epithelial injury. *International Journal of Molecular Medicine*.

[21] Kim J. H., Park D. J., Yun J. C. (2012). Human adipose tissue-derived mesenchymal stem cells protect kidneys from cisplatin nephrotoxicity in rats. *American Journal of Physiology-Renal Physiology*.

[22] Magnasco A., Corselli M., Bertelli R. (2008). Mesenchymal stem cells protective effect in adriamycin model of nephropathy. *Cell Transplantation*.

[23] Moustafa F. E., Sobh M. A., Abouelkheir M. (2016). Study of the effect of route of administration of mesenchymal stem cells on cisplatin-induced acute kidney injury in Sprague Dawley rats. *International Journal of Stem Cells*.

[24] Putra A., Pertiwi D., Milla M. N. (2019). Hypoxia-preconditioned MSCs have superior effect in ameliorating renal function on acute renal failure animal model. *Open Access Macedonian Journal of Medical Sciences*.

[25] Qian H., Yang H., Xu W. (2008). Bone marrow mesenchymal stem cells ameliorate rat acute renal failure by differentiation into renal tubular epithelial-like cells. *International Journal of Molecular Medicine*.

[26] Rashed L. A., Elattar S., Eltablawy N., Ashour H., Mahmoud L. M., El-Esawy Y. (2018). Mesenchymal stem cells pretreated with melatonin ameliorate kidney functions in a rat model of diabetic nephropathy. *Biochemistry and Cell Biology*.

[27] Sarhan M., El Serougy H., Hussein A. M. (2014). Impact of bone-marrow-derived mesenchymal stem cells on adriamycin-induced chronic nephropathy. *Canadian journal of physiology and pharmacology*.

[28] Selim R. E., Ahmed H. H., Abd-Allah S. H. (2019). Mesenchymal stem cells: a promising therapeutic tool for acute kidney injury. *Applied Biochemistry and Biotechnology*.

[29] Sun J. H., Teng G. J., Ma Z. L., Ju S. H. (2008). In vivo monitoring of magnetically labeled mesenchymal stem cells administered intravascularly in rat acute renal failure. *Swiss Medical Weekly*.

[30] Zickri M. B., Zaghloul S., Farouk M., Fattah M. M. (2012). Effect of stem cell therapy on adriamycin induced tubulointerstitial injury. *International Journal of Stem Cells*.

[31] Mata-Miranda M. M., Bernal-Barquero C. E., Martinez-Cuazitl A. (2019). Nephroprotective effect of embryonic stem cells reducing lipid peroxidation in kidney injury induced by cisplatin. *Oxidative Medicine and Cellular Longevity*.

[32] Vazquez-Zapien G. J., Martinez-Cuazitl A., Rangel-Cova L. S., Camacho-Ibarra A., Mata-Miranda M. M. (2019). Biochemical and histological effects of embryonic stem cells in a mouse model of renal failure. *Romanian journal of morphology and embryology*.

[33] Minocha E., Sinha R. A., Jain M., Chaturvedi C. P., Nityanand S. (2019). Amniotic fluid stem cells ameliorate cisplatin-induced acute renal failure through induction of autophagy and inhibition of apoptosis. *Stem Cell Research & Therapy*.

[34] Sun B., Luo X., Yang C. (2019). Therapeutic effects of human urine-derived stem cells in a rat model of cisplatin-induced acute kidney injury in vivo and in vitro. *Stem Cells International*.

[35] Zhang C., George S. K., Wu R. (2020). Reno-protection of urine-derived stem cells in a chronic kidney disease rat model induced by renal ischemia and nephrotoxicity. *International Journal of Biological Sciences*.

[36] Sun W., Zhu Q., Yan L., Shao F. (2019). Mesenchymal stem cells alleviate acute kidney injury via miR-107-mediated regulation of ribosomal protein S19. *Annals of Translational Medicine*.

[37] Foroutan T., Nafar M., Motamedi E. (2020). Intraperitoneal injection of graphene oxide nanoparticle accelerates stem cell therapy effects on acute kidney injury. *Stem Cells Cloning*.

[38] Zhou T., Liao C., Li H. Y., Lin W., Lin S., Zhong H. (2020). Efficacy of mesenchymal stem cells in animal models of lupus nephritis: a meta-analysis. *Stem Cell Research & Therapy*.

[39] Chen Y., Tang X., Li P. (2019). Bone marrow derived mesenchymal stromal cells ameliorate ischemia/reperfusion injury-induced acute kidney injury in rats via secreting tumor necrosis factor-inducible gene 6 protein. *BioMed Research International*.

[40] Xiu G.-H., Zhou X., Li X.-L. (2018). Role of bone marrow mesenchymal stromal cells in attenuating inflammatory reaction in lipopolysaccaride-induced acute kidney injury of rats associated with TLR4-NF-kappa B signaling pathway inhibition. *Annals of Clinical & Laboratory Science*.

[41] Tan J., Wu W., Xu X. (2012). Induction therapy with autologous mesenchymal stem cells in living-related kidney transplants: a randomized controlled trial. *JAMA*.

[42] Vanikar A. V., Trivedi H. L., Kumar A. (2014). Co-infusion of donor adipose tissue-derived mesenchymal and hematopoietic stem cells helps safe minimization of immunosuppression in renal transplantation - single center experience. *Renal Failure*.

[43] Zhou T., Liao C., Lin S., Lin W., Zhong H., Huang S. (2020). The efficacy of mesenchymal stem cells in therapy of acute kidney injury induced by ischemia-reperfusion in animal models. *Stem Cells International*.

[44] Zhou T., Li H. Y., Liao C., Lin W., Lin S. (2020). Clinical efficacy and safety of mesenchymal stem cells for systemic lupus erythematosus. *Stem Cells International*.

[45] El-Metwaly S., El-Senduny F. F., El-Demerdash R. S., Abdel-Aziz A. F. (2019). Mesenchymal stem cells alleviate hydrochloric acid-induced lung injury through suppression of inflammation, oxidative stress and apoptosis in comparison to moxifloxacin and sildenafil. *Heliyon*.

[46] Li P., Chen Y., Yang K., Chen D., Kong D. (2020). Mechanical characteristics of BMSCs-intervened sciatic nerve in chronic alcohol-intoxicated animal model. *International Journal of Neuroscience*.

[47] Liu B., Ding F.-X., Liu Y. (2018). Human umbilical cord-derived mesenchymal stem cells conditioned medium attenuate interstitial fibrosis and stimulate the repair of tubular epithelial cells in an irreversible model of unilateral ureteral obstruction. *Nephrology*.

[48] Togel F. E., Westenfelder C. (2010). Mesenchymal stem cells: a new therapeutic tool for AKI. *Nature reviews. Nephrology*.

[49] Barnes C. J., Distaso C. T., Spitz K. M., Verdun V. A., Haramati A. (2016). Comparison of stem cell therapies for acute kidney injury. *American Journal of Stem Cells*.

[50] Kale S., Karihaloo A., Clark P. R., Kashgarian M., Krause D. S., Cantley L. G. (2003). Bone marrow stem cells contribute to repair of the ischemically injured renal tubule. *Journal of Clinical Investigation*.

[51] Togel F., Hu Z., Weiss K., Isaac J., Lange C., Westenfelder C. (2005). Administered mesenchymal stem cells protect against ischemic acute renal failure through differentiation-independent mechanisms. *American Journal of Physiology-Renal Physiology*.

[52] Wang Z., Li S., Wang Y., Zhang X., Chen L., Sun D. (2019). GDNF enhances the anti-inflammatory effect of human adipose-derived mesenchymal stem cell-based therapy in renal interstitial fibrosis. *Stem Cell Research*.

